# Measurement of serum AST activity using the Seralyzer system

**DOI:** 10.1155/S1463924685000451

**Published:** 1985

**Authors:** J. F. Stevens, R. G. Newall

**Affiliations:** 1 Division of Pathology St. Stephen's Hospital Chelsea London SWIO 9TH UK; 2 Miles Laboratories Ltd Stoke Court Stoke Poges Buckinghamshire SL2 4LY UK


**Journal of Automatic Chemistry**/*Journal of Clinical Laboratory Automation*, **Vol. 7, No. 4 (October–December 1985), pp. 204–205**

### Measurement of serum AST activity using the Seralyzer system

**J. F. Stevens**
*Division of Pathology, St. Stephen's Hospital, Chelsea, London SW10 9TH*

**and R. G. Newall**
*Miles Laboratories Ltd, Stoke Court, Stoke Poges, Buckinghamshire SL2 4LY, UK*

The performance of the Seralyzer reflectance photometer system [1] for the measurement of various analytes [2 and 3] and for the enzymes CK and LDH [4] has been previously reported by the authors. This paper describes an evaluation of a new assay for AST (Aspartate Transaminase) determination.

The Seralyzer AST system (Ames Division) is based on the measurement of the rate of formation of a quinone-imine dye at 530 nm at a temperature of 37 °C, with an incubation and test period of 4 min. Serum specimens are diluted 1 in 3 prior to analysis; for AST activities above 250 IU/L a further nine-fold dilution is required. The reaction sequence is shown below:

$$\text{Aspartate} + \alpha\text{-Ketoglutarate} \xrightarrow{\text{AST}} \text{Glutamate} + \text{Oxaloacetate} \xrightarrow[\text{Oxaloacetate decarboxylase}]{Mg^{++}} \text{Pyruvate} + CO_2$$

$$\text{Pyruvate} \xrightarrow[\text{Pi,TPP},Mg^{++}]{\text{Pyruvate oxidase}} \text{Acetyl-phosphate} + H_2O_2 + CO_2 \xrightarrow[\text{DHBS, 4-Aminoantipyrine}]{\text{Peroxidase}} \text{Quinoneimine dye} + H_2O$$

Comparisons were made with the Centrifichem (Baker) ultra-violet method using Boehringer reagents supplied for this system (BCL), also performed at 37 °C. Normal and high control sera (pooled normal control sera, and Dade II control serum, respectively) were used for precision measurements, while 183 routine hospital clinical serum specimens were analysed in duplicate by both methods. Total bilirubin estimations were also made.

*Table 1. Analytical precision of Seralyzer and Centrifichem AST methods—using control sera, and duplicate testing of patient sera.*

| | Between-run | | Within-run |
|---|---|---|---|
| | Low control | High control | Patient sera duplicates |
| Seralyzer: | | | |
| Mean (IU/L) | 36·4 | 130·7 | 86·9 |
| S.D. | 5·2 | 7·6 | 1·66 |
| Centrifichem: | | | |
| Mean (IU/L) | 23·3 | 98·3 | 81·5 |
| S.D. | 2·5 | 2·5 | 1·78 |
| *N* | 23 | 23 | 183 |

The between-run and within-run precision figures were obtained from the control sera included in each batch of analyses, and from the patient sera analysed in duplicate, respectively (table 1). The within-run precision showed no difference between the Seralyzer and the Centrifichem (SD 1.66 and 1.78 IU/l respectively). The between-run precision showed a better performance for the Centrifichem than the Seralyzer (SD 2·5 as opposed to 6·4) and this probably reflects the smaller batches used with the Seralyzer requiring a calibration for each batch—for the purpose of this study only. In practice, the Seralyzer calibration would not be redone unless the batch of strips were changed or the control values were out of range. The precision was acceptable for clinical purposes. When comparing the two systems for accuracy (table 2), the Seralyzer and the Centrifichem showed a good correlation over the range 0–250 IU/l ($r$ = 0·95, slope = 0·95). Samples with values over 250 IU/l on the Seralyzer are repeated in dilution, and the correlation over the whole range was equally good ($r$ = 0·93, slope = 0·95).

*Table 2. Linear regression comparison of accuracy of Seralyzer and Centrifichem AST results, using clinical serum specimens.*

| AST range | *N* | Intercept | Slope | Correlation coefficient |
|---|---|---|---|---|
| 0–250 | 170 | 7·83 | 0·95 | 0·95 |
| 0–700 | 183 | 7·97 | 0·95 | 0·93 |

The results from the Seralyzer on patient sample were on average 6·7% higher than the Centrifichem values. The differences in the control sera were greater, but this difference is probably due to a matrix effect affecting the two methods differently.

Values for control sera and normal ranges should, therefore, be determined for the Seralyzer AST method before routine use of the system.

In many analyses, bilirubin is said to be a possible interfering substance, and for this reason total bilirubin was measured at the same time as the AST levels. Although the bilirubin values covered the range 0–280 μmol/l, no direct effect of bilirubin on either method could be demonstrated.

In summary, a rapid (4 min) AST method is now available, which is simple to use by the trained operator, and which demonstrates performance essentially equivalent to conventional high-cost, automated laboratory methodology. The Seralyzer AST system would be especially suitable for emergency or out-of-hours estimations in this routine hospital laboratory, particularly where this is accompanied by rapid CK measurements on the same system.

### References

1. Zipp, A., *Journal of Automatic Chemistry*, **3** (1981), 71.

2. Stevens, J. F. and Newall, R. G., *Journal of Clinical Pathology*, **36** (1983), 9.

3. Stevens, J. F., Tsang, W. and Newall, R. G., *Journal of Clinical Pathology*, **36** (1983), 598.

4. Stevens, J. F., Tsang, W. and Newall, R. G., *Journal of Clinical Pathology*, **36** (1983), 1371.

### HPLC '86: THE TENTH INTERNATIONAL SYMPOSIUM ON COLUMN LIQUID CHROMATOGRAPHY

To be held at the Sheraton Palace Hotel, San Francisco, USA from 18 to 23 May 1986.

Chaired by Dr Ronald E. Majors of Varian Associates, HPLC '86 has the following sponsors:

San Francisco Bay Area Chromatography Colloquium
Federation of Chromatography Discussion Groups
Chromatography Sub-Division of the American Society
Japanese Society of Analytical Chemistry and Agencies
American Society for Testing Materials E-19 on Chromatography
Arbeitskreis Chromatographie der Fachgruppe
Chemie der Gesellschaft Deutscher Chemiker
The Chromatographic Society (UK)
Groupement pour l'Advancement des Méthodes et Physico-Chimiques d'Analyse (G.A.M.S.)
Österreichische Gesellschaft für Mikrochemie und Chemie
Analytical Chemistry Divisions of the Koninkliijke Chemische Vereniging
Schweizerischer Chemiker-Verband
Societe Chimique de France
Svenska Kemistsamfundet

The scientific program will include numerous oral and poster papers on the latest HPLC applications, theory, columns, and instrumentation. Special lecture sessions will be devoted to specific topics such as the use of HPLC in biotechnology, techniques of optimization of methods development, new detection possibilities, and micro LC columns.

Discussion sessions will serve to augment the formal presentations and allow the participants to take an active part in exploring solutions to their chromatography problems, the future potential of HPLC, and the exchange of ideas.

Poster papers will again be a mainstay of HPLC '86. These poster sessions are always popular because they give interested participants the opportunity to spend more time with the authors on an individual or small group basis in order to discuss the subtleties and 'how to's' of their contributions.

Poster sessions will not be scheduled opposite lecture sessions to provide sufficient time for poster viewing and discussion.

*Exhibition*

In conjunction with the Symposium, an exhibition of the latest liquid chromatography instrumentation, columns, and accessories is planned. Booth space is limited and early response has been advised.

*Further details from Shirley Schlessinger, Symposium Manager, 400 E. Randolph Drive, Chicago, Illinois 60601, USA.*

